# GPNN: Power studies and applications of a neural network method for detecting gene-gene interactions in studies of human disease

**DOI:** 10.1186/1471-2105-7-39

**Published:** 2006-01-25

**Authors:** Alison A Motsinger, Stephen L Lee, George Mellick, Marylyn D Ritchie

**Affiliations:** 1 Center for Human Genetics Research and Department of Molecular Physiology and Biophysics, Vanderbilt University Medical School, Nashville, TN, 37232-0700, USA; 2 Dartmouth Medical School, One Medical Center Drive, Lebanon, New Hampshire 03756-001, USA; 3 University of Queensland, School of Medicine and Department of Neurology, Princess Alexandra Hospital, Brisbane, Australia

## Abstract

**Background:**

The identification and characterization of genes that influence the risk of common, complex multifactorial disease primarily through interactions with other genes and environmental factors remains a statistical and computational challenge in genetic epidemiology. We have previously introduced a genetic programming optimized neural network (GPNN) as a method for optimizing the architecture of a neural network to improve the identification of gene combinations associated with disease risk. The goal of this study was to evaluate the power of GPNN for identifying high-order gene-gene interactions. We were also interested in applying GPNN to a real data analysis in Parkinson's disease.

**Results:**

We show that GPNN has high power to detect even relatively small genetic effects (2–3% heritability) in simulated data models involving two and three locus interactions. The limits of detection were reached under conditions with very small heritability (<1%) or when interactions involved more than three loci. We tested GPNN on a real dataset comprised of Parkinson's disease cases and controls and found a two locus interaction between the *DLST *gene and sex.

**Conclusion:**

These results indicate that GPNN may be a useful pattern recognition approach for detecting gene-gene and gene-environment interactions.

## Background

One goal of genetic epidemiology is to identify polymorphisms associated with common, complex multifactorial diseases. Success in achieving this goal will depend on a research strategy that recognizes and addresses the importance of interactions among multiple genetic and environmental factors in the etiology of diseases such as essential hypertension [1-3]. One traditional approach to modeling the relationship between discrete predictors such as genotypes and discrete clinical outcomes is logistic regression [4]. Logistic regression is a parametric statistical approach for relating one or more independent or explanatory variables (e.g. polymorphisms) to a dependent or outcome variable (e.g. disease status) that follows a binomial distribution. However, as reviewed by Moore and Williams [2], the number of possible interaction terms grows exponentially as each additional main effect is included in the logistic regression model. Thus, logistic regression is limited in its ability to deal with interactions involving many factors. Having too many independent variables in relation to the number of observed outcome events is a well-recognized problem [5,6] and is an example of the curse of dimensionality [7]. In response to this limitation, Ritchie et al. [8] developed a genetic programming optimized neural network (GPNN). Neural networks are a class of pattern recognition methods developed in the 1940's to model the neuron, the basic functional unit of the brain [9]. A major advantage of neural networks in comparison to traditional analysis approaches is their ability to take what is learned on a given dataset about the relationship between independent variables and an outcome variable and make predictions on data where the outcome variable is unknown [10]. One disadvantage of neural networks is that the network architecture must be pre-specified and there is no rule of thumb for generating this architecture. Thus, trial and error processes often take place [11]. GPNN was developed in an attempt to improve upon the trial-and-error process of choosing an optimal architecture for a pure feed-forward back propagation neural network. The GPNN optimizes the inputs from a larger pool of variables, the weights, and the connectivity of the network including the number of hidden layers and the number of nodes in the hidden layer. Thus, the algorithm attempts to generate optimal neural network architecture for a given data set. This is an advantage over the traditional back propagation NN in which the inputs and architecture are pre-specified and only the weights are optimized.

Parkinson's disease (PD) is a debilitating neurodegenerative disorder characterized clinically by progressive rigidity, tremor, bradykinesia (slowness of movement), and postural instability [12]. PD affects approximately 2% of the population over the age of 65, increasing to approximately 5% of the population by the age of 85 [13]. PD is characterized pathologically by widespread neurodegeneration, especially of the dopaminergic cells of the substantia nigra pars compacta. The cause of this neurodegeneration is unknown, but is hypothesized to result from complex interactions between genetic and environmental factors affecting energy metabolism and protein turnover. Mellick and colleagues previously investigated single nucleotide polymorphisms (SNPs) in the mitochondrial complex I as potential genetic susceptibility factors for PD. They investigated 70 SNPs in 31 nuclear complex I genes in 306 PD patients and 321 controls. No evidence for a single locus association was identified [12], but a two-factor gene-environment interaction between the *DLST *gene and sex was detected using Multifactor Dimensionality Reduction (MDR) [14], another methodology for detecting epistatic interactions.

Although previous empirical studies suggest GPNN is a useful method for identifying gene-gene interactions [8], the power of GPNN for high-order gene-gene interaction models is not known and its application to real datasets has not been reported. The goal of the present study was to evaluate the power of GPNN for identifying gene-gene interactions using simulated data representing a variety of epistasis models, and to test this methodology on an actual dataset of SNPs in mitochondrial complex I nuclear encoded genes in Parkinson's disease cases and controls.

## Results

The results of the simulation study are shown in Tables 1, 2, and 3. Here, we list the 40 epistasis models sorted by allele frequency and number of loci along the vertical axis and heritability across the horizontal axis. Table 1 shows the results for a sample size of 200 cases and 200 controls. Table 2 shows the results for a sample size of 400 cases and 400 controls. Table 3 shows the results for a sample size of 800 cases and 800 controls. For the sample size of 400 total individuals, in the two locus models, GPNN had greater than 94% power for all heritability values greater than 1.5%, greater than 77% for heritability of 1%, and much lower power for the very small genetic effect of 0.5%. In the three locus models the power of GPNN was greater than 94% in the 0.2/0.8 allele frequency models with greater than 2% heritability. However, it was much lower in the 0.4/0.6 allele frequency models as well as all heritability values of 1.5% and lower. In the four and five locus models, the power of GPNN was very low for all heritability values. For the sample size of 800 total individuals, in the two locus models, GPNN had greater than 65% power for the epistasis models evaluated at all heritability levels. In the three, four and five locus models, the trend is similar to the smaller sample size. In the total sample size of 1600 individuals, GPNN has greater than 86% power in the two locus models. Again, the three, four, and five locus models showed similar trends. However, in general, the larger number of individuals did increase the power to detect the functional loci.

**Figure 1:**
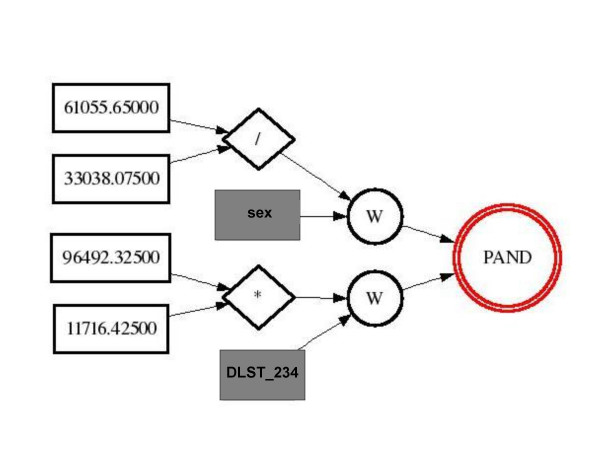
**GPNN model for Parkinson's Disease data**. A GPNN model that was evolved by GPNN on the PD data. The real numbers are used to create weights and fill in for the W nodes. The individual values of sex and *DLST*_234 fill into those nodes. The activation function is a Boolean function AND, thus it will take (61055.5/33038.075)*sex AND (96492.325*11716.425)*DLST_234.

The results of the real data analysis are shown in Table 4. Here, we show the GPNN model selected from each cross-validation interval. This includes the factors included in the final model for each interval, as well as the classification error and prediction error of the model. As can be seen in the distribution of factors in the model, *DLST_234 *and *sex *are the most commonly detected factors. This two-factor model was selected and used to fit a final GPNN model. This model correctly predicts PD status 59.66% of the time (p < 0.001). The GPNN model that describes this interaction is shown in Figure 1.

The results of the stepwise logistic regression (LR) analysis of the PD data are shown in Table 5. These results are similar to the GPNN results however, LR selected additional loci. The final LR model included *sex, DLST_234, FA1_897*, and *FA10_200*. Because the results included additional loci, we implemented a forward selection LR using only the variables identified by GPNN including the interaction term (as described in the methods section). The results of the forward selection logistic regression using *sex, DLST_234*, and the interaction term yielded similar results for *DLST *and sex, shown in Table 6. The interaction term of *sex *and *DLST_234 *was not statistically significant.

## Discussion

Identifying disease susceptibility genes associated with common complex, multifactorial diseases is a major challenge for genetic epidemiology. One of the dominating factors in this challenge is the difficulty of detecting gene-gene interactions with currently available statistical approaches. To deal with this issue, new statistical approaches have been developed such as GPNN. GPNN has been shown to have higher power than a back propagation NN using simulated data generated under five two-locus epistasis models [8].

The goal of the current study was to evaluate the power of GPNN for detecting high-order gene-gene interactions using simulated data representing a variety of epistasis models. Based on the results shown in Table 1, 2, and 3, there is an obvious trend in the power to detect the gene-gene interactions in these simulated data. With a sample size of 400 individuals (200 cases and 200 controls), GPNN has high power to detect interactions in two locus models for all heritability values tested with the exception of 0.5% (which is a very small genetic effect). As the number of interacting loci increases or the heritability decreases, there is a decrease in the power of GPNN. With a sample size of 800 or 1600 individuals (400 cases, 400 controls or 800 cases, 800 controls), GPNN has high power to detect interactions in all two locus models evaluated. Similar to the trend seen in the sample size of 400 individuals, there is a decrease in power as the number of interacting loci increases. There is, however, an increase in power in the data with the larger sample size. We explored the limits of GPNN in terms of genetic effect (heritability), sample size, and number of interacting loci. Using simulated data models involving two and three locus interactions, we show that GPNN has high power to detect gene-gene interactions in models with very small heritability values (2–3% heritability) that are well within the range of most common complex diseases in simulated data models. The limits of detection were reached under conditions with very small heritability (<1%) or when interactions involved more than three loci. For example, Alzheimer's disease is estimated to have heritability between 60–75% [15] while breast, colorectal, and prostate cancers are 27%, 35%, and 42% respectively [16]. Effects as small as those simulated would be very difficult to detect using any method.

Secondly, the sample size was held constant at either 400, 800, or 1600 individuals. These sample sizes may be too small for detection of high-order interaction models. If you consider a two-locus interaction model, there are nine two-locus genotype combinations for the cases and controls to be distributed. When you extend this to a three-locus model, there are 27 genotype combinations. This sample of individuals continues to be distributed in the four and five locus models with 81 and 243 genotype combinations respectively. This demonstrates that the 400, 800, or 1600 individuals are then distributed much more sparsely across the genotype combinations. This is an example of the curse of dimensionality [7]. Therefore, to detect gene-gene interactions composed of greater than three loci, the sample size may need to be substantially larger than 400 cases and 400 controls. This is an active area of further study to determine if there is a direct relationship between sample size and number of interacting loci. If a direct relationship exists, this can be used for performing sample size and power calculations for real data analyses rather than performing empirical power studies in the future.

Our analysis has revealed an interesting mitochondrial gene-sex interaction leading to altered risk for PD. Substantial evidence directly links mitochondrial dysfunction to Parkinsonism. For example, mutations in the *PINK1 *gene (which codes for a mitochondrial kinase) lead to rare autosomal recessive forms of PD [17]. Moreover, mitochondrial toxins such as MPTP and rotenone can induce Parkinsonism in animals and humans [18]. In addition, mtDNA polymorphisms have also been suggested to influence risk for sporadic PD. The recent study of van der Walt and colleagues [19] revealed an association between a non-conservative amino acid changing mtDNA SNP in the ND3 gene and reduced risk for PD. Interestingly, this decreased risk appeared to be stronger in women than men. Gender differences in the incidence of PD are well documented with men at 1.5 times greater risk [20]. There are many possible reasons for this, including gender specific gene-environment interactions.

Our model correctly predicts PD status 59.66% of the time (p < 0.001). Because we can only predict ~60% of the individuals' disease status, it is clear that many additional etiological factors are involved. It is important to note that the nature of the effect detected cannot be fully elucidated from this analysis. Based on the logistic regression analyses, we confirm that *DLST_234 *and *sex *are statistically significant however, the interaction term is not. This could indicate that the effect is not a multiplicative interaction. It could also indicate that logistic regression is underpowered for detecting an interaction of this magnitude in a sample size of approximately 600 individuals. Biologically, it remains uncertain how the *DLST_234 *SNP-gender interaction, uncovered in the current analysis, might influence risk for PD. The dihydrolipoyl succinyl transferase (*DLST*) gene codes for one of three components of the thiamine-dependent mitochondrial alpha-ketoglutarate complex. The *DLST_234 *SNP is located in an intron at the 3' end of the *DLST *gene and is unlikely to have a biological impact on the function of this gene. Because SNPs across the *DLST *locus have been shown to exhibit strong linkage disequilibrium however, it is more likely that the *DLST_234 *SNP represents other genetic variability within this locus. Further work is required to determine how genetic variation in this locus may influence PD. As is the case for all initial case-control associations, it is important to consider the possibility that this finding is a result of idiosyncrasies in one isolated data set. Interestingly, this finding was detected despite a relatively small sample size of approximately 300 cases and 300 controls. Moreover, this interaction was detected using MDR, an independent approach for detecting gene-gene interactions [14]. It will be important to replicate this study using other datasets with larger sample sizes or consisting of different populations. Nonetheless, our results clearly demonstrate the application of this innovative method to probe for interactive effects in real data sets.

While these results demonstrate the lower limits of GPNN's power to detect gene-gene interactions, there are still many more questions to be addressed. First, it will be important to extend the simulation studies to include larger sample sizes and a larger range of higher heritability values. Secondly, while GPNN has good power to detect gene-gene interactions, the robustness of the method in the presence of error has not been evaluated. Thus, a simulation study including data with genotyping error, phenocopy, genetic heterogeneity, and missing data may provide more insight into the robustness. Finally, a larger set of epistasis models including those with a small degree of main effect as well as a significantly higher number of SNPs in the study would provide further evidence of the power of GPNN.

Since most common diseases have heritability greater than 20%, and GPNN was shown to have 100% power for heritability of 5% [8], GPNN should have high power for detecting interactions in most common diseases. GPNN is likely to be a powerful pattern recognition approach for the detection of gene-gene interactions in future studies of common human disease.

## Conclusion

A genetic programming neural network (GPNN) is a novel pattern recognition approach for detecting gene-gene and gene-environment interactions in studies of human disease. GPNN has high power to detect two and three-locus interactions in moderate sample sizes. Higher order interactions will require larger sample sizes. In addition, GPNN detected a gene-sex interaction associated with Parkinson's disease. GPNN, and similar methods, will be useful in dissecting the genetic architecture of common, complex disease.

## Methods

### A Genetic Programming Neural Network Approach

**Figure 2:**
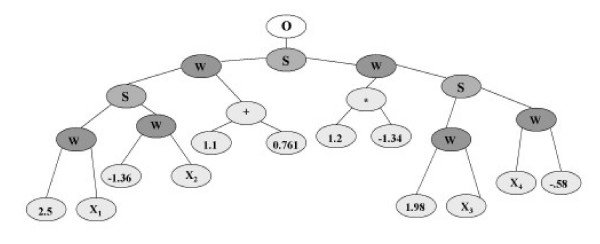
**A NN evolved by GPNN**. An example of a NN evolved by GPNN. The O is the output node, S indicates the activation function, W indicates a weight, and X_1_-X_4 _are the NN inputs.

GPNN was developed to improve upon the trial-and-error process of choosing an optimal architecture for a pure feed-forward back propagation neural network (NN) [8]. Optimization of NN architecture using genetic programming (GP) was first proposed by Koza and Rice [21]. The goal of this approach is to use the evolutionary features of genetic programming to evolve the architecture of a NN. The use of binary expression trees allow for the flexibility of the GP to evolve a tree-like structure that adheres to the components of a NN. Figure 2 shows an example of a binary expression tree representation of a NN generated by GPNN. The GP is constrained such that it uses standard GP operators but retains the typical structure of a feed-forward NN. A set of rules is defined prior to network evolution to ensure that the GP tree maintains a structure that represents a NN. The rules used for this GPNN implementation are consistent with those described by Koza and Rice [21]. The flexibility of the GPNN allows optimal network architectures to be generated that consist of the appropriate inputs, connections, and weights for a given data set.

**Figure 3:**
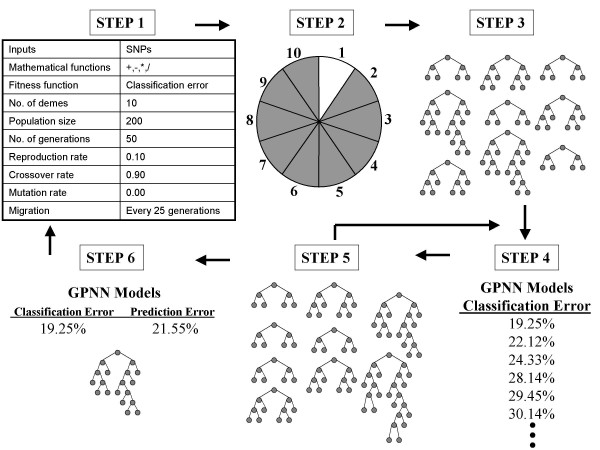
Overview of GPNN Method.

The GPNN method has been described in detail [8]. The steps of the GPNN method are shown in Figure 3 and described in brief as follows. First, GPNN has a set of parameters that must be initialized before beginning the evolution of NN models including the operating parameters of the GP. Second, the data are divided into 10 equal parts for 10-fold cross-validation. Cross-validation is an important statistical technique in model building that allows one to develop generalizable models and prevent over-fitting of data. The goal is to identify a model that predicts well in an independent data set. Cross-validation provides one with a training data set to use in model building as well as a test data set to use for model validation. Thus, in ten-fold cross-validation, 9/10 of the data are used for training and 1/10 of the data are used for testing. Further detail regarding the implementation of cross-validation can be found in [22]. Cross-validation was selecting rather than a training-testing-validation approach because cross-validation allows one to maximize the use of all data in the process. The train-test-validation sampling approach reduces the effective sample size of each set. In addition, cross-validation provides an unbiased estimate of the statistic being used to assess the data [23].

Third, training of the GPNN begins by generating an initial random population of solutions. Each solution is a binary expression tree representation of a NN, similar to that shown in Figure 2. Fourth, each GPNN is evaluated on the training set and its fitness recorded. Fifth, the best solutions are selected for crossover and reproduction using a fitness-proportionate selection technique, called roulette wheel selection, based on the classification error of the training data [24]. Classification error is defined as the proportion of individuals where the disease status was incorrectly specified. A predefined proportion of the best solutions will be directly copied (reproduced) into the new generation. Another proportion of the solutions will be used for crossover with other best solutions. The new generation, which is equal in size to the original population, begins the cycle again. This continues until some criterion is met at which point GPNN stops. Sixth, this best GPNN model is tested on the 1/10 of the data left out to estimate the prediction error of the model. Prediction error is a measure of the ability to predict case or control status in the 1/10 of the data. Steps two through six are performed ten times with the same parameters settings, each time using a different 9/10 of the data for training and 1/10 of the data for testing.

The results of a GPNN analysis include 10 GPNN models, one for each split of the data. In addition, a classification error and prediction error is recorded for each of the models. A cross-validation consistency can be measured to determine those variables which have a strong signal in the gene-gene interaction model [8,25-27]. Cross-validation consistency is the number of times a particular combination of variables are present in the GPNN model out of the ten cross-validation data splits. Thus a high cross-validation consistency, ~10, would indicate a strong signal, whereas a low cross-validation consistency, ~1, would indicate a weak signal and a potentially false positive result. The loci combination with the highest cross-validation consistency is chosen as the final model.

### Data simulation

The goal of the simulation study was to generate data sets that exhibit gene-gene interactions for the purpose of evaluating the power of GPNN. We simulated a collection of models with varying heritability, allele frequency, and number of interacting polymorphisms. Additionally, we used a constant sample size for all simulations. We selected the sample size of 200 cases and 200 controls because this is a typical sample that is used in many epidemiology studies. We also extended this to 400 cases and 400 controls as well as 800 cases and 800 controls.

As discussed by Templeton [28], epistasis, or gene-gene interaction, occurs when the combined effect of two or more genes on a phenotype could not have been predicted from their independent effects. It is anticipated that epistasis is likely to be a ubiquitous component of the genetic architecture of common human diseases [3]. Current statistical approaches in human genetics focus primarily on detecting the main effects and rarely consider the possibility of interactions [28]. In contrast, we are interested in simulating data using different epistasis models that exhibit minimal independent main effects, but produce an association with disease primarily through interactions. In this study, we use penetrance functions as genetic models. Penetrance functions model the relationship between genetic variations and disease risk. Penetrance is defined as the probability of disease given a particular combination of genotypes.

To evaluate the power of GPNN for detecting gene-gene interactions, we simulated case-control data using a variety of epistasis models in which the functional loci are single-nucleotide polymorphisms (SNPs). We selected models that exhibit interaction effects in the absence of any main effects. Interactions without main effects are desirable because they provide a high degree of complexity to challenge the ability of a method to identify gene-gene interactions.

To generate a variety of epistasis models for this study, we selected three criteria for variation. First, we selected two different allele frequencies. An allele frequency of 0.8/0.2 was selected so that we could evaluate the ability of GPNN in situations where there is a relatively rare allele. In addition, the frequency of 0.6/0.4 was selected to allow for the situation where both alleles are relatively common. Second, we selected a range of epistatic heritability values including 3%, 2%, 1.5%, 1%, and 0.5%. These heritability values fall into the realm of very small genetic effects. We chose to simulate data using epistasis models with such small heritability values to test the lower limits of GPNN. Finally, we selected epistasis models with a varying number of interacting loci of two, three, four, or five. We speculate that common diseases will be comprised of complex interactions among many loci. The number of interacting loci simulated here may still be too few to be biologically relevant. However, no gene-gene interaction models exist beyond the five locus level at this time.

We generated models using software described by Moore et al. [29]. We selected models from all possible combinations of allele frequency, heritability, and number of loci. This resulted in 40 total models. Each data set consisted of either 200 cases and 200 controls, 400 cases and 400 controls, or 800 cases and 800 controls and a gene-gene interaction model comprised of two, three, four, or five functional interacting SNPs. We simulated 100 data sets of each model consisting of the functional SNPs and either eight non-functional SNPs for the two, three, and four SNP models or ten non-functional SNPs for the five SNP models. This resulted in 12,000 total datasets. We used a dummy variable encoding for the genotypes where *n-1 *dummy variables are used for *n *levels [30]. Based on the dummy coding, these data would have either 20 or 24 variables for the two-four SNP models or five SNP models respectively. All data sets are available from the authors upon request.

### PD case-control sample

Full methodological details for the case-control data have been published previously [12]. In brief demographic characteristics are shown in Table 7 and described as follows. PD patients (n = 305) were recruited from hospitals, private neurological clinics, and PD support groups from throughout Queensland, Australia. Control subjects (n = 321) consisted of healthy spouses of affected PD patients and other unaffected volunteers collected from patient neighborhoods and communities. Seventy (70) SNPs corresponding to nuclear-coded mitochondrial complex I genes were genotyped using the dynamic allele specific hybridization (DASH) method. Twenty-two SNPs were polymorphic in the study population and included in the final association study. A list of these polymorphisms can be found in Table 8.

### Data analysis

We used GPNN to analyze 100 data sets for each of the epistasis models. The GP parameters settings for GPNN included 10 demes, migration every 25 generations, population size of 200 per deme, 50 generations, crossover rate of 0.90, and a reproduction rate of 0.10. GPNN is not required to use all the variables as inputs. Here, GPNN performed random variable selection in the initial population of solutions. Through evolution, GPNN selects those variables that are most relevant. We calculated a cross-validation consistency for each data set. This measure is defined as the number of times each SNP is in the GPNN model across the ten cross-validation intervals. Thus, one would expect a strong signal to be consistent across all ten or most of the data splits, where a false positive signal may be present in only one or a few of the cross-validation intervals. We estimated the power of GPNN as the number of times the correct functional SNPs had a cross-validation consistency that was higher than all other SNPs in the dataset, divided by the total number of datasets for each epistasis model. Either one or both of the dummy variables could be selected to consider a locus present in the model. We used the same GPNN parameters for the real data analysis.

We also conducted a stepwise logistic regression analysis to determine what solution a logistic regression modeling procedure would detect. We conducted the regression analysis in SAS v9.1. We used p < 0.20 for inclusion in the model and p < 0.10 for remaining in the model. This results in a list of all candidate genes with statistically significant main effects. We also tested the model including the variables detected by GPNN (sex and *DLST*_234, and the interaction term) using forward selection. The same inclusion criteria (p < 0.20) was implemented.

## Authors' contributions

AAM and MDR participated in the design of the study, statistical analyses, and writing of the manuscript. GM participated in the design and coordination of the PD study and preparation of the final draft of the manuscript. SL participated in discussions regarding PD results and preparation of the final draft of the manuscript. All authors read and approved the final manuscript.

## Figures and Tables

**Table 1:** GPNN Power (%) Results – Sample Size 400

		Heritability				
Allele freq	Number loci	3%	2%	1.5%	1%	0.5%

.2/.8	2	100	94	97	81	24
.4/.6	2	100	99	99	77	16
.2/.8	3	99	94	22	4	3
.4/.6	3	75	35	20	3	1
.2/.8	4	46	23	0	5	0
.4/.6	4	11	2	0	2	0
.2/.8	5	0	1	0	1	0
.4/.6	5	0	0	0	0	0

**Table 2:** GPNN Power (%) Results – Sample Size 800

		Heritability				
Allele freq	Number loci	3%	2%	1.5%	1%	0.5%

.2/.8	2	100	100	100	99	76
.4/.6	2	100	100	100	99	65
.2/.8	3	98	100	31	10	12
.4/.6	3	97	50	42	15	3
.2/.8	4	68	42	4	14	2
.4/.6	4	34	11	3	3	1
.2/.8	5	2	6	0	6	0
.4/.6	5	1	0	0	1	0

**Table 3:** GPNN Power (%) Results – Sample Size 1600

		Heritability				
Allele freq	Number loci	3%	2%	1.5%	1%	0.5%

.2/.8	2	100	97	100	100	97
.4/.6	2	100	100	100	99	86
.2/.8	3	100	99	40	21	20
.4/.6	3	97	65	53	20	6
.2/.8	4	70	45	15	11	3
.4/.6	4	30	15	5	3	0
.2/.8	5	2	1	0	0	0
.4/.6	5	2	0	0	0	0

**Table 4:** GPNN Results from Parkinson's Disease Data Analysis

CV	Factors in Model							CE	PE
1	*FB4_5152*	*sex*						0.4050	0.4127
2	*DLST_234*	*sex*						0.3978	0.5079
3	*DLST_234*	*sex*						0.3996	0.3810
4	*FA6_5146*	*FB7_5144*	*FS8_5155*	*FV2_0182*	*sex*			0.3936	0.4355
5	*FS7_5156*	*sex*						0.4007	0.4355
6	*FA7_5148*	*FB9_5142*	*FS1_5158*	*FS4_5133*	*sex*			0.3989	0.3871
7	*DLST_234*	*FA7_5148*	*sex*	*sex*				0.3989	0.3871
8	*DLST_234*	*FV2_0182*	*FV2_0182*	*sex*	*sex*			0.3828	0.5323
9	*DLST_234*	*DLST_234*	*FS7_5156*	*FS8_5155*	*FV2_0182*	*sex*	*sex*	0.3982	0.4098
10	*DLST_234*	*DLST_234*	*FA6_5146*	*FS4_5133*	*sex*			0.3929	0.3934

**Table 5:** Stepwise Logistic Regression Results from Parkinson's Disease Analysis

Effect	Point Estimate	p-value	OR	95% Wald CI	
*DLST_234*	0.2501	0.0438	1.284	1.007	1.638
*FA1_8197*	0.3908	0.0491	1.478	1.002	2.181
*FA10_200*	-0.1879	0.0913	0.829	0.666	1.031
*Sex_M1*	-0.7997	<.0001	0.449	0.324	0.623

**Table 6:** Forward Logistic Regression Results from Parkinson's Disease Analysis

Effect	Point Estimate	p-value	OR	95% Wald CI	
*DLST_234*	0.2564	0.0374	1.292	1.015	1.645
*Sex_M1*	-0.7730	<.0001	0.462	0.334	0.638

**Table 7:** Demographic characteristics

	Cases (n = 305)	Controls (n = 321)	p-value
Sex	166 males	206 males	<.0001*
	139 females	115 females	
Average age	67 ± 9 years	65 ± 9 years	0.0131**
Average age of onset	60 ± 10 years	NA	

* based on Fisher's exact test

** based on Student's t-test

**Table 8:** List of mitochondrial polymorphisms

Marker	dbSNP ID#	Major Allele Frequency
*DLST_234(A/G)*	rs1799900	A = 51.4
*FA1_5157(G/C)*	rs1801316	G = 98.7
*FA1_8196(T/C)*	rs1800823	T = 92.9
*FA1_8197(T/G)*	rs1800824	T = 92.9
*FA6_5146(C/T)*	rs1801311	C = 66.4
*FA7_5148(C/T)*	rs1045629	C = 83.0
*FA8_5147(A/G)*	rs4679	A = 58.8
*FA8_8968(G/A)*	rs6822	G = 87.7
*FA10_151(G/A)*	ss10349	G = 83.0
*FA10_200(A/G)*	ss16204	A = 62.0
*FB4_5152(C/T)*	rs12762	C = 89.3
*FB7_5144(C/G)*	rs9543	C = 53.2
*FB8_5127(C/A)*	rs1800662	C = 77.9
*FB9_5142(C/T)*	rs1128560	C = 96.1
*FS1_5158(G/T)*	rs1801317	G = 54.3
*FS1_5159(A/G)*	ss2421568	A = 64.8
*FS2_1886(T/A)*	rs12570	T = 66.9
*FS4_5133(A/G)*	rs567	A = 50.5
*FS4_5178(G/A)*	rs31303	G = 77.3
*FS7_5156(T/C)*	rs1801315	T = 57.1
*FS8_5155(C/T)*	rs1051806	C = 80.7
*FV2_0182(C/T)*	rs906807	C = 81.3
